# Supplementary figures and images for: Chitosan primes plant defence mechanisms against Botrytis cinerea, including expression of Avr9/Cf‐9 rapidly elicited genes
Source: Plant Cell Environ. 2020 Nov 4;44(1):290–303. doi: 10.1111/pce.13921 (PMC7821246; doi:10.1111/pce.13921)

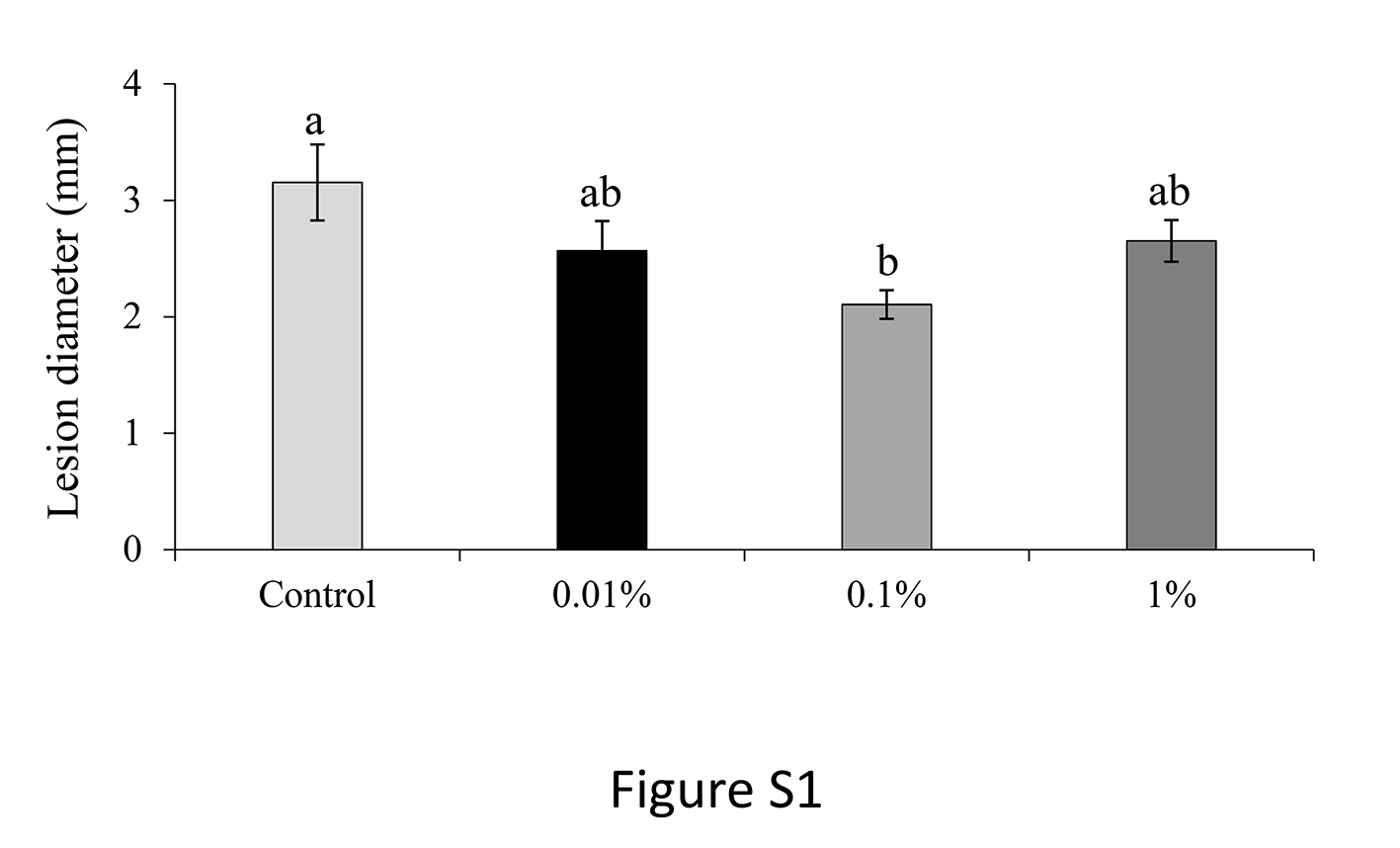

Supplement: Supplementary file 1 — FIGURE S1 Chitosan‐induced resistance in Solanaceae melongena (aubergine). Disease lesions at 3 dpi. Values represent means ± SEM (n = 10). Different letters indicate statistically significant differences among treatments (least significant differences, α = 0.05) [file PCE-44-290-s001.tif]

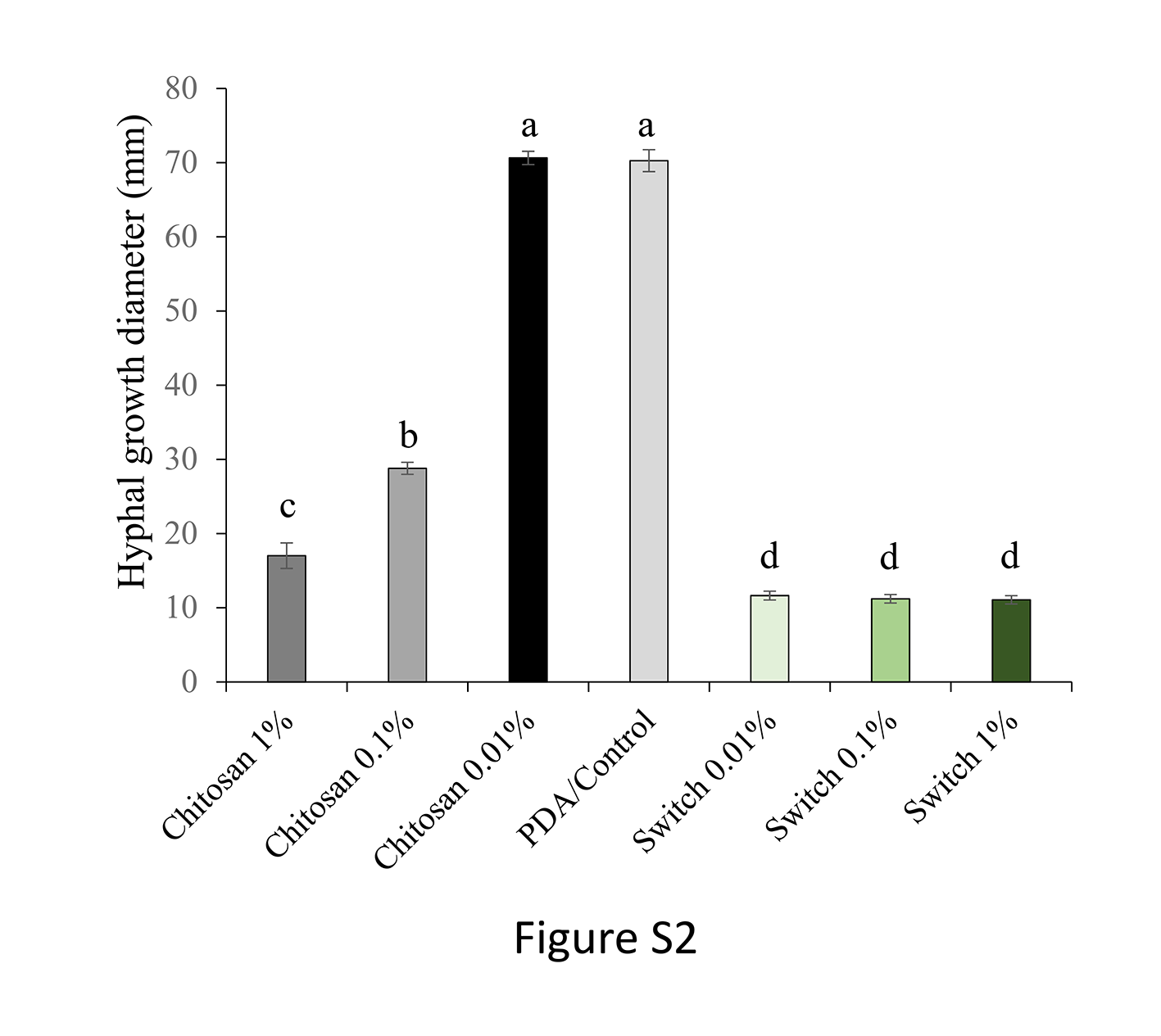

Supplement: Supplementary file 2 — FIGURE S2 Chitosan and Switch fungicide antifungal activity against Botrytis cinerea. Bars represent means of fungal growth diameter (±SEM, n = 5) at 4 days after inoculating PDA‐containing Petri dishes with 5 mm agar plugs of actively growing B. cinerea mycelia. Different letters indicate statistically significant differences among treatments (least significant differences, α = 0.05) [file PCE-44-290-s002.tif]

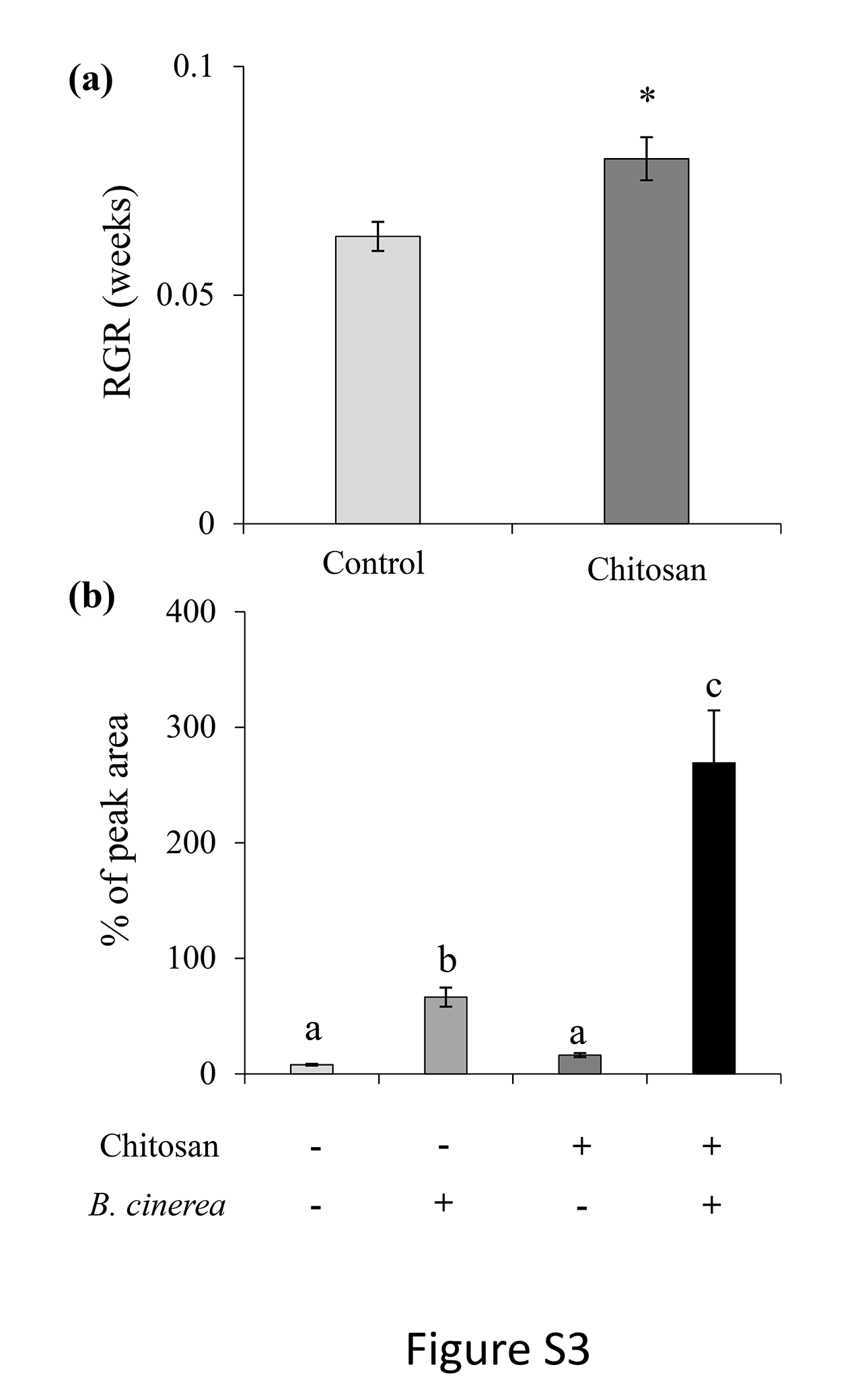

Supplement: Supplementary file 3 — FIGURE S3 Chitosan‐induced resistance is based on defence priming. (A) Relative growth rate (RGR) per week of tomato plants 1 and 2 weeks after treatment with 0.01% chitosan. Values represent means ± SEM (n = 10). Asterisk indicates statistically significant differences among treatments (Student's T. test, α = 0.05). (B) Mass‐spectrometry quantification (% of peak area) of Jasmonic acid‐isoleucine (JA‐ile) at 24‐h post inoculation. Values represent means ± SEM (n = 4). Different letters indicate statistically significant differences among treatments (least significant differences, α = 0.05) [file PCE-44-290-s003.tif]

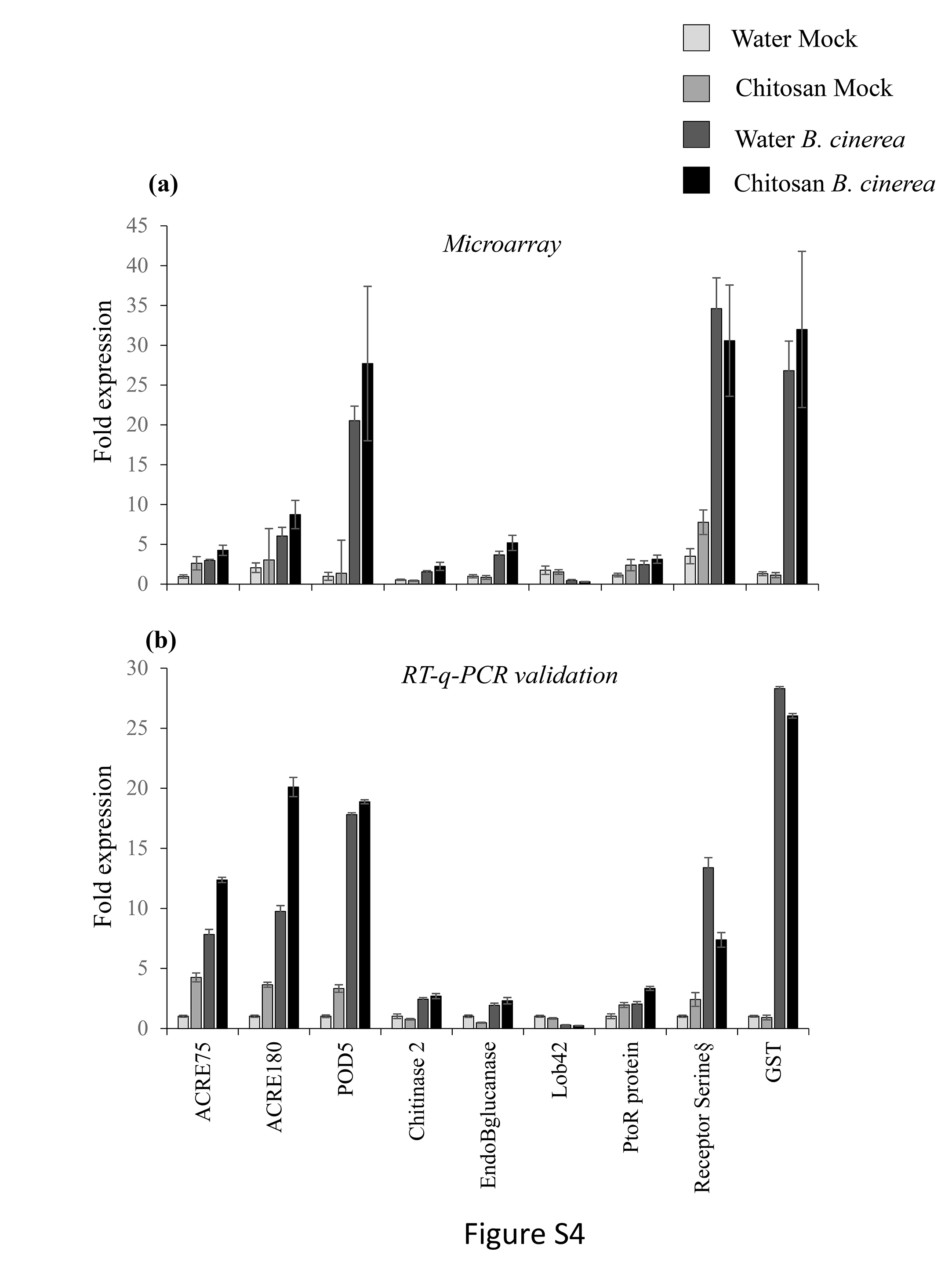

Supplement: Supplementary file 4 — FIGURE S4 Validation of microarray expression results. Expression profile obtained in the microarray (A) and in the analysis by RT‐q‐PCR (B) of a subset of nine genes at 9 hpi with Botrytis cinerea [file PCE-44-290-s004.tif]

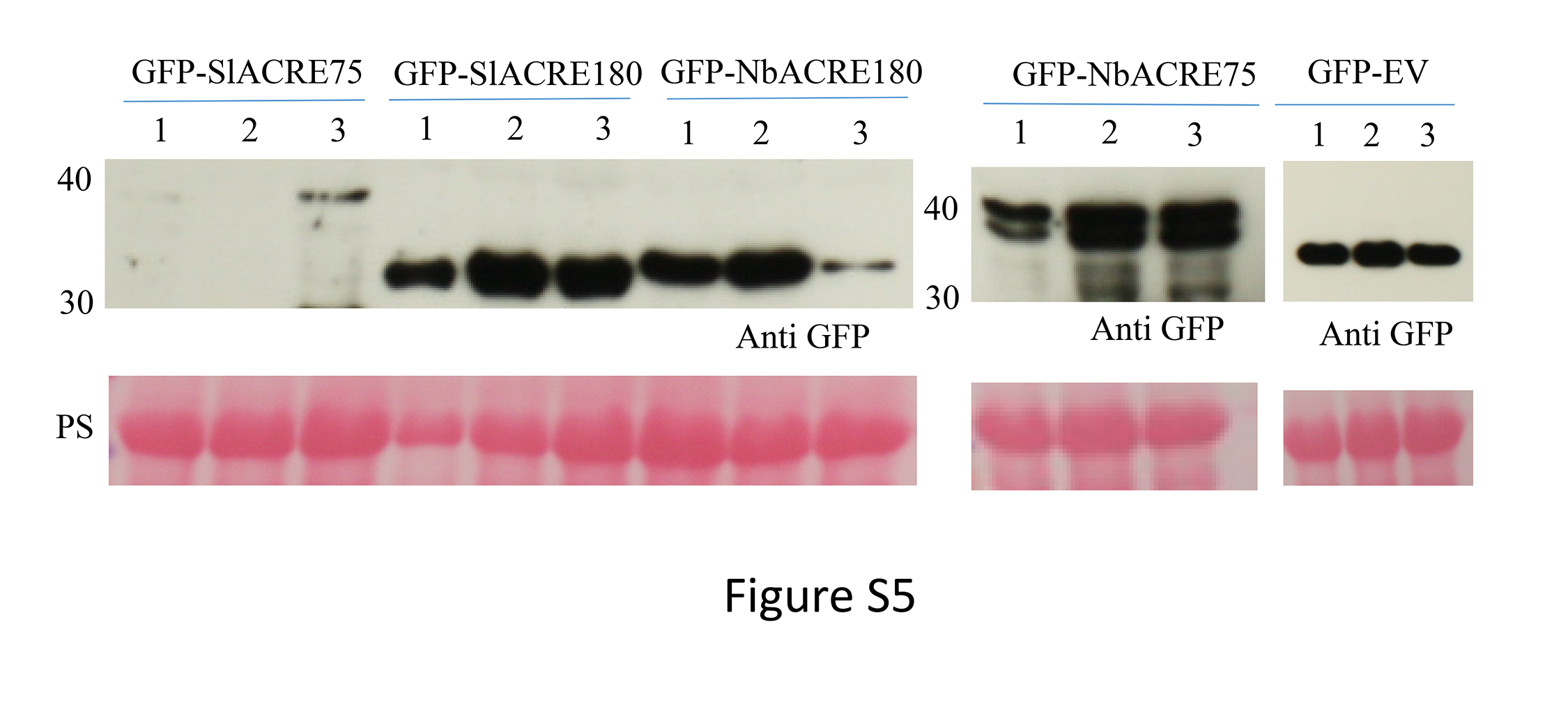

Supplement: Supplementary file 5 — FIGURE S5 Western Blot analysis. Expression of proteins by immunoblot analysis of GFP‐SlACRE75, GFP‐SlACRE180, GFP‐NbACRE75 and GFP‐NbACRE180 fusion proteins in N. benthamiana leaves at 48 h after agroinfiltration. Expected protein sizes were (i) SlACRE75 = 14.79 + 26 KDa GFP = 40.8 KDa; (ii) SlACRE180 = 10.86 + 26 = 36.8 KDa; (iii) NbACRE180 = 11.74 + 26 = 37.7 KDa; and (iv) NbACRE75 = 14.6 + 26 = 40.7 KDa. Proteins were separated by SDS–PAGE and analyzed by immunoblotting. A GFP‐specific antibody was used for detection of GFP‐fusion protein. Equal loading of total proteins was examined by Ponceau staining (PS). Three lanes represent three replicates per construct GFP‐SlACRE75, GFP‐SlACRE180, GFP‐NbACRE75, GFP‐NbACRE180 and a GFP‐non‐protein/empty vector (control) [file PCE-44-290-s005.tif]

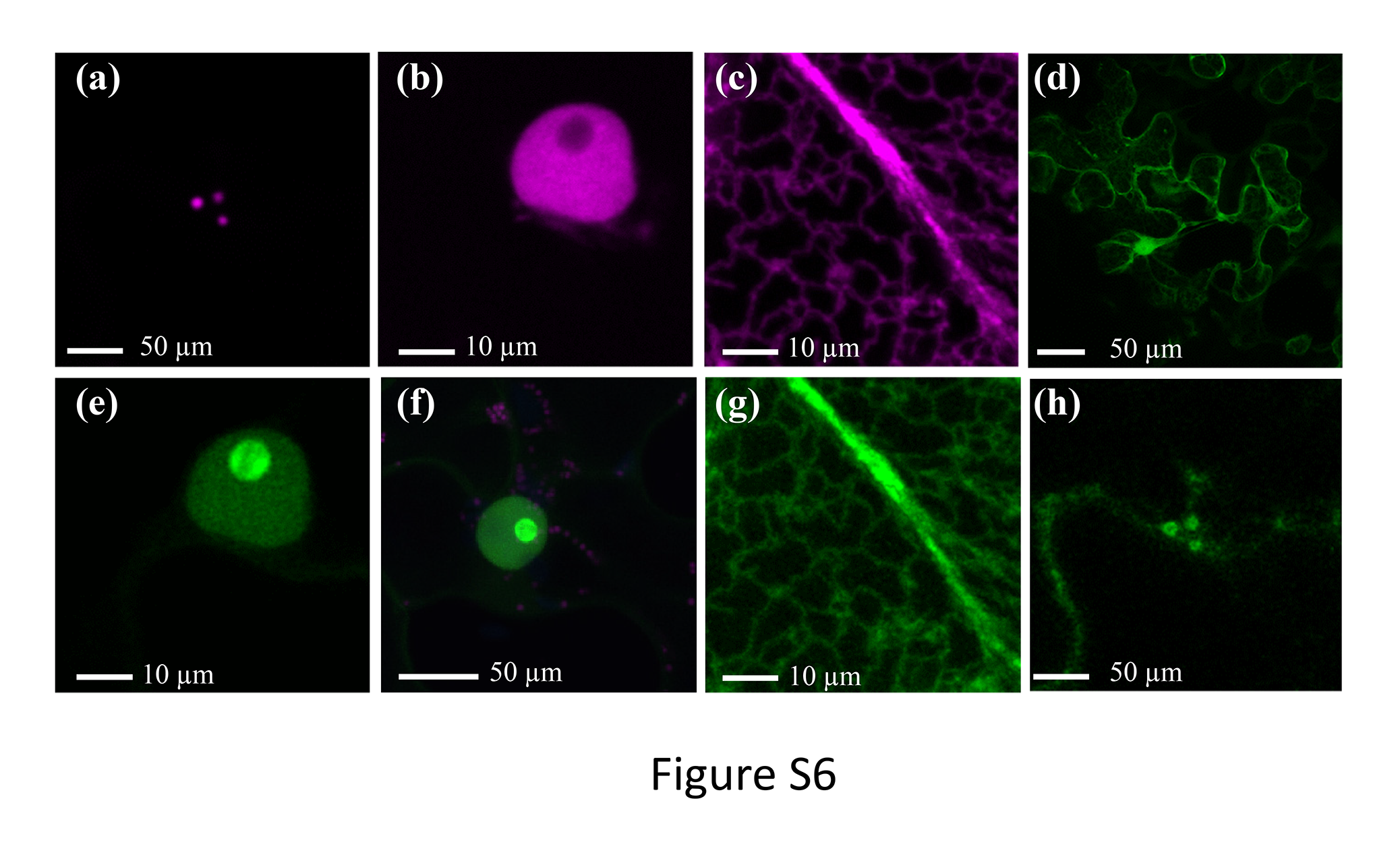

Supplement: Supplementary file 6 — FIGURE S6 Subcellular location of ACRE proteins. Confocal microscopy observation of (A) pFlub vector as a RFP‐peroxisome tagged marker, (B) nucleus mRFP marker, (C) ER mRFP marker, (D) free GFP in cytoplasm and the nucleus, (E) GFP‐SlACRE75 and (F) GFP‐NbACRE75 fusions in the nucleus and nucleolus, (G) GFP‐SlACRE180 fusion in the ER and (H) GFP‐NbACRE180 fusion in the peroxisomes [file PCE-44-290-s006.tif]

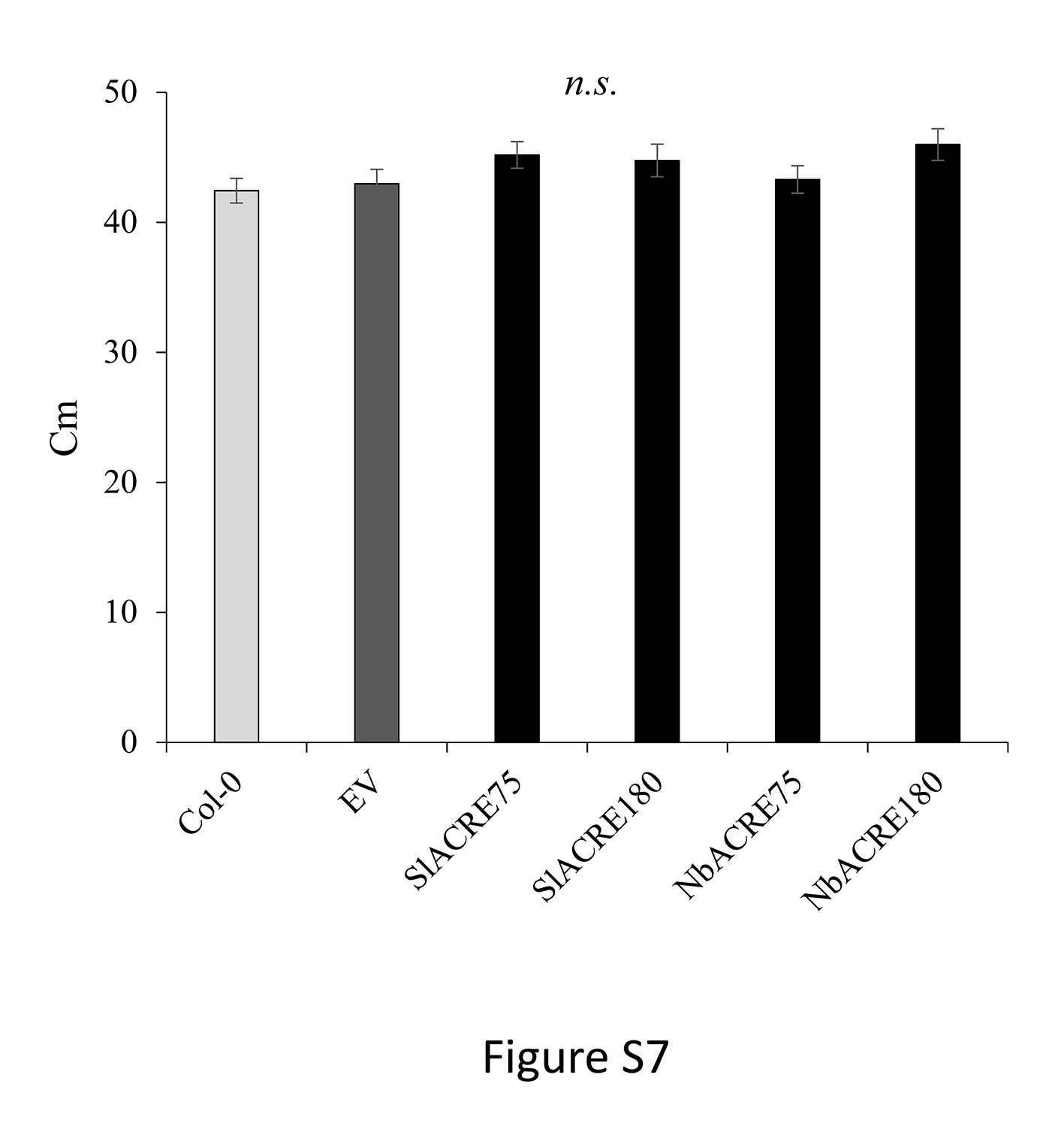

Supplement: Supplementary file 7 — FIGURE S7 Growth analysis. Perimeter in cm of rosettes from Arabidopsis lines overexpressing GFP‐Empty vector (EV), GFP‐SlACRE75, GFP‐SlACRE180, GFP‐NbACRE75 and GFP‐NbACRE180 constructs representing biomass. Values represent means ± SEM (n = 8–16). n.s, not significant differences between treatments (One‐way ANOVA, α = 0.05) [file PCE-44-290-s007.tif]
